# Host Genetic Background Effect on Body Weight Changes Influenced by Heterozygous *Smad*4 Knockout Using Collaborative Cross Mouse Population

**DOI:** 10.3390/ijms242216136

**Published:** 2023-11-09

**Authors:** Nayrouz Qahaz, Iqbal M. Lone, Aya Khadija, Aya Ghnaim, Osayd Zohud, Nadav Ben Nun, Aysar Nashef, Imad Abu El-Naaj, Fuad A. Iraqi

**Affiliations:** 1 Department of Clinical Microbiology and Immunology, Sackler Faculty of Medicine, Tel-Aviv University, Tel Aviv 69978, Israel; nayrouz.qahaz@gmail.com (N.Q.); iqbalzoo84@gmail.com (I.M.L.); aya.khadeja91@gmail.com (A.K.); ayaghnaim89@gmail.com (A.G.); osaydzohud@gmail.com (O.Z.); nadavbennun1@mail.tau.ac.il (N.B.N.); 2 Department of Oral and Maxillofacial Surgery, Baruch Padeh Medical Center, Poriya 15208, Israel; dr.aysarn@gmail.com (A.N.); iabu@poria.health.gov.il (I.A.E.-N.)

**Keywords:** Collaborative Cross (CC), Smad4 knockout, body weight gain, machine learning (ML), genotyping

## Abstract

Obesity and its attendant conditions have become major health problems worldwide, and obesity is currently ranked as the fifth most common cause of death globally. Complex environmental and genetic factors are causes of the current obesity epidemic. Diet, lifestyle, chemical exposure, and other confounding factors are difficult to manage in humans. The mice model is helpful in researching genetic BW gain because genetic and environmental risk factors can be controlled in mice. Studies in mouse strains with various genetic backgrounds and established genetic structures provide unparalleled opportunities to find and analyze trait-related genomic loci. In this study, we used the Collaborative Cross (CC), a large panel of recombinant inbred mouse strains, to present a predictive study using heterozygous *Smad*4 knockout profiles of CC mice to understand and effectively identify predispositions to body weight gain. Male C57Bl/6J Smad4+/− mice were mated with female mice from 10 different CC lines to create F1 mice (Smad4+/−x CC). Body weight (BW) was measured weekly until week 16 and then monthly until the end of the study (week 48). The heritability (H2) of the assessed traits was estimated and presented. Comparative analysis of various machine learning algorithms for predicting the BW changes and genotype of mice was conducted. Our data showed that the body weight records of F1 mice with different CC lines differed between wild-type and mutant Smad4 mice during the experiment. Genetic background affects weight gain and some lines gained more weight in the presence of heterozygous Smad4 knockout, while others gained less, but, in general, the mutation caused overweight mice, except for a few lines. In both control and mutant groups, female %BW had a higher heritability (H2) value than males. Additionally, both sexes with wild-type genotypes showed higher heritability values than the mutant group. Logistic regression provides the most accurate mouse genotype predictions using machine learning. We plan to validate the proposed method on more CC lines and mice per line to expand the literature on machine learning for BW prediction.

## 1. Introduction

Obesity and its attendant conditions have become major health problems worldwide and obesity is currently ranked as the fifth most common cause of death globally [1]. Over the last few years, the prevalence of obesity has steadily increased and almost tripled since 1975. Complex environmental and genetic factors are causes of the current obesity epidemic. The unparalleled availability of inexpensive, appealing, calorie-dense foods, coupled with a decline in occupational physical activity, has created a setting conducive to obesity. Despite this, a substantial proportion of the world’s population remains lean, indicating the existence of genetic factors that determine an individual’s tendency to develop obesity. This genetic predisposition to obesity is determined by the interplay of numerous risk genes, each of which has a negligible influence on its own [2,3].

Numerous chronic disorders, including hypertension, dyslipidemia, metabolic syndrome, type 2 diabetes (T2D), cardiovascular disease (CVD), non-alcoholic fatty liver disease, Alzheimer’s disease, and cancer, are associated with obesity [4,5]. Being overweight/obese is a substantial contributor to the increased incidence and prevalence of cancer and may surpass smoking as the most important avoidable cause of cancer [6]. The cessation of tobacco use and reductions in overweight and obesity rates may be the most influential lifestyle changes affecting human health and cancer in particular.

Obesity is associated with a greatly higher risk of several malignant carcinomas [7,8,9] and contributes to a poor prognosis and survival rate. The required processes and mechanisms for clarifying this relationship are poorly understood. Reducing the prevalence of obesity should be the ultimate goal. Therefore, population-wide education regarding a healthy lifestyle must be intensified. However, education alone is not sufficient to optimize the weight of the entire population. To minimize the risk of obesity-related disorders, including metabolic syndrome and cancer susceptibility, it is also necessary to investigate druggable targets for weight reduction. Hopefully, better knowledge of the role of genetic functions in regulating body weight will result in the creation of novel diagnostic and treatment methods.

GWAS research has related several loci and genes to illnesses and phenotypic features, including body weight, obesity, and neurological disorders [10,11]. Life history and genetic/environmental combinations often determine disease risk [12,13,14]. Diet, lifestyle, chemical exposure, and other confounding factors are difficult to manage in humans. The mice model is useful for researching genetics of BW gain because genetic and environmental risk factors can be controlled in mice. Studies in mouse strains with various genetic backgrounds and established genetic structures provide unparalleled opportunities to find and analyze trait-related genomic loci.

Smad4 was first identified as a tumor suppressor in pancreatic cancer and later as a TGF-mediator [15,16]. Smad4 plays a key role in TGF-/BMP signaling by forming complexes with receptor-activated Smads: Smad2 and 3 or Smad1 and 5. Somatic inactivation of Smad4 is a frequent event in multiple tumor types. Moreover, Smad4 deletion in murine tissues, in combination with other genetic alterations that cause tumor initiation, resulted in cancer lesions of the colon [17,18], pancreas [19,20], stomach [21], and liver [22]. Additionally, Smad4 correlated with depth of invasion and pathologic stage as well as regional metastases and decreased survival [23]. In animal studies, global deletion of Smad2 or Smad4 is lethal, whereas Smad3-deficient mice are viable [24]. Consequently, TGF-1/Smad3 signaling has received a great deal of attention. Mice lacking Smad3 are resistant to high-fat diet (HFD)-induced obesity, insulin resistance, and diabetes in numerous models [25,26]. In contrast, little is known about Smad4 in obesity or diabetes [27]. Still, its absence in endothelial cells was reported to result in a significant reduction in the beiging process, leading to diminished expression of beige fat markers like UCP1 and Tbx14. Furthermore, Smad4 facilitates angiogenesis during beiging, promoting the development of capillary structures and increased expression of angiogenic genes. These findings suggest that Smad4 is a key regulator of beige fat induction and vascular development within white adipose tissue [28].

In this study, we used a mouse panel formed by crossing Smad4+/−mice with Collaborative Cross (CC) mice [29], i.e., mice from a highly genetically diverse collection of recombinant inbred lines (RILs), which allows the timely and cost-efficient mapping of target regions as quantitative trait loci (QTL) responsible for the genetic variance of a specific complex trait [30]. The CC strains are derived from the crossing of eight genetically diverse founder strains (A/J, C57BL/6J, 129S1/SvImJ, NOD/LtJ, NZO/HiLtJ, CAST/EiJ, PWK/PhJ, and WSB/EiJ). Three founders of the CC strain (CAST/EiJ, PWK/PhJ, and WSB/EiJ) are wild-derived, and five of them are inbred lines [31]. The development of the Collaborative Cross (CC) mouse model involved a unique breeding approach designed to randomize the genetic composition of each inbred line. This innovative strategy was devised to create recombinant CC inbred lines that represent the genomes of the eight founders of CC mice. In this approach, the eight founding strains are arranged in different positions within the breeding process, ensuring that each CC line originates from an independent breeding funnel of the eight CC founders. As a result, each recombination site in the CC population is uniquely generated, contributing to the remarkable genetic diversity of this model. The CC mouse population was shown to be the most powerful genetic reference population (GRP) for studying and dissecting complex traits compared to other reported approaches [32,33,34,35]. The key features of the CC are its relation to gene mapping through providing a very large number of segregated genetic variants in the population (there are over 36 million SNPs) [36] and its relatively high level of recombination compared to other mouse RI sets. The CC mouse GRP provides a unique platform and resource for studying BW complexity associated with obesity and BW gain influenced by the Smad4 knockout gene.

Finally, we applied machine learning (ML) methods to our recorded data to classify the different genetic backgrounds under the assessed traits and to provide us with a tool to predict outcomes based on earlier data. ML is a field of study that uses computational algorithms to turn empirical data into models for the purpose of analysis. It allows for the strengthening of statistical and computational approaches that auto-generate given data [37]. Recently, ML methods have been implemented in several disease recognition and other health-related applications [1]. However, there is a dearth of literature exploring the links between ML methods and weight gain. Furthermore, it is critical to identify the potential relationship with the genetic background of obesity; however, this has often been overlooked in prior studies, particularly in mouse models.

## 2. Results

### 2.1. Generation of F1 (Smad4x CC) Mice

In this study, we assessed 391 F1 mice of 10 different CC lines, an average of 39.2 mice per line. The mice were assigned to four different experimental groups, as was described earlier. Briefly, we maintained 391 F1 mice, males, and females, with mutant and wild-type Smad4 genotypes for 48 weeks (Table 1), with an average of 9.8 mice per group. Approximately half were heterozygous for the knockout allele (51.9% versus 48.1% in the control group), and 51.7% were female versus 48.3% male (Table 2). 

### 2.2. Dynamics of Body Weight (BW) Changes during the Experimental Period for F1 (Smad4x CC) Mice

Figure S1 shows the BW measurements of 10 F1 CC-C57BL/6-Smad4 lines at 14 time points. At 6 weeks, the control mice weighed from 19.09 to 21.53 g (Figure S1b), while F1 CC-Smad4+/−mice weighed from 17.43 to 21.72 g (Figure S1b). Smad4+/− lines had significant (*p* < 0.05) body weight differences. Except for IL2513, the control and Smad4+/− groups had no initial differences. Both groups and lines gained weight during the trial. Only males from line 6018 began losing weight around week 32 in both control and mutant groups, as shown in (Figure S2). Females reached a near plateau at weeks 40 and 48, with a modest increase (Figure S2a). IL2513 weighed the least, while IL6012 weighed the most (Figure S2), even in Figure S1, which shows the dynamic BW for each line regardless of sex.

**Figure 1:**
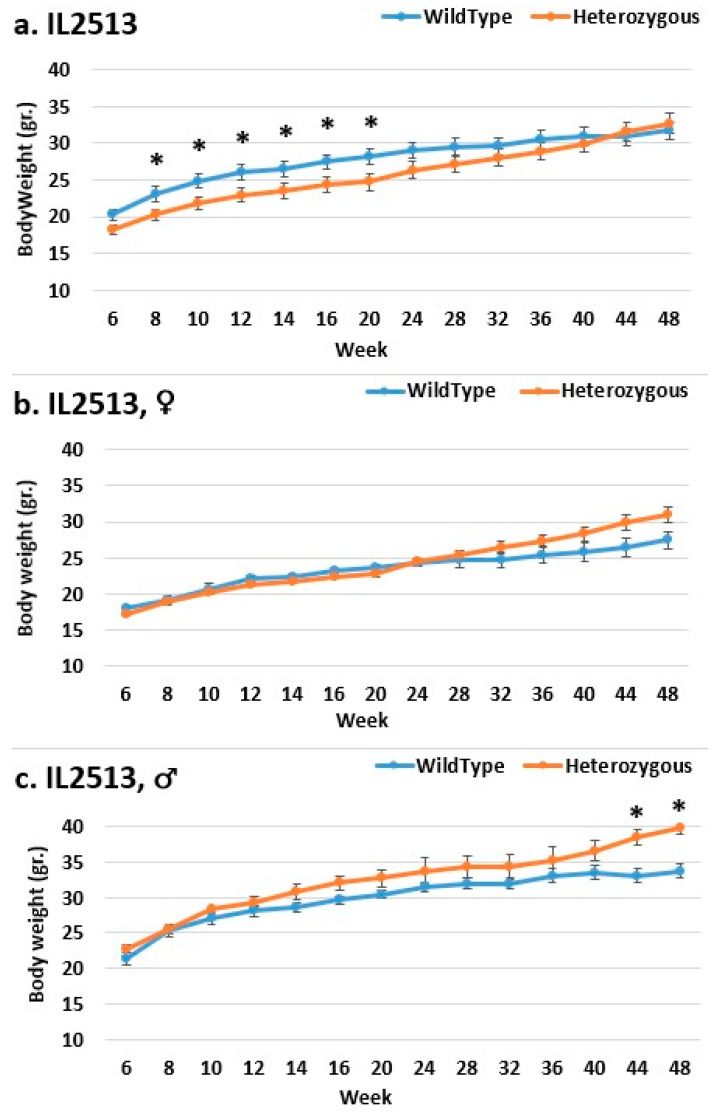
Body weight changes in wild-type and Smad4+/− mice at 14 time points for line IL2513 (6–48 weeks old). The X-axis depicts time points (in weeks), while the y-axis depicts body weight values. The weight increase trend in this line is distinctive and distinct from that of the other lines; in the overall group (**a**), the weight of control mice is greater than the weight of mutant mice up to a reversal point at which the weight ratio reverses, occurring at 44 weeks. The difference is significant between control mice and mutant mice BW from the start of the experiment to an age of 20 weeks in the overall group. In the female group (**b**), the bending point begins at the 20-week-old reversal point and grows somewhat until the end of the experiment, but not significantly. There is no bending point in males (**c**). Mutant mice weighed more than control mice from the start of the experiment, but the difference in the weights grew with time and became significant only around weeks 44 and 48. * shows a significant variation between the two lines (values) at the specific time point.

The control lines’ BWs ranged from 31.6750 (IL2513) to 48.3063 (IL6012) at the end of the experiment. F1 Smad4+/− carriers weighed 32.7–50.31g. Both control and Smad4+/− carriers had significantly different final body weights (Figure 1). 

### 2.3. Dynamics of BW Changes in Grams over the 48-Week Experiment for Each CC Line 

Each CC mouse line has a distinct pattern of BW gain in both control and mutant animals, and the weight difference between control and mutant mice is unique to each line. Except for lines IL2513, IL2750, IL3348, and IL5000, mutant mice in most lines and all analysis groups of each line weighed more than control mice. Line IL2513 has a unique weight gain pattern; in the overall group, control mice were heavier than mutant mice until the weight ratio reversed, which occurred at 44 weeks (Figure 1). From the start of the experiment to week 20, the difference in BW between control and mutant mice is significant. In the female group, the reversal point occurs at week 20, and the weight difference increases, but not significantly, during the experiment. Males have no bending point; mutant mice weighed more than control mice from the start of the experiment, but the weight difference grew over time and became significant only between weeks 44 and 48.

**Figure 2:**
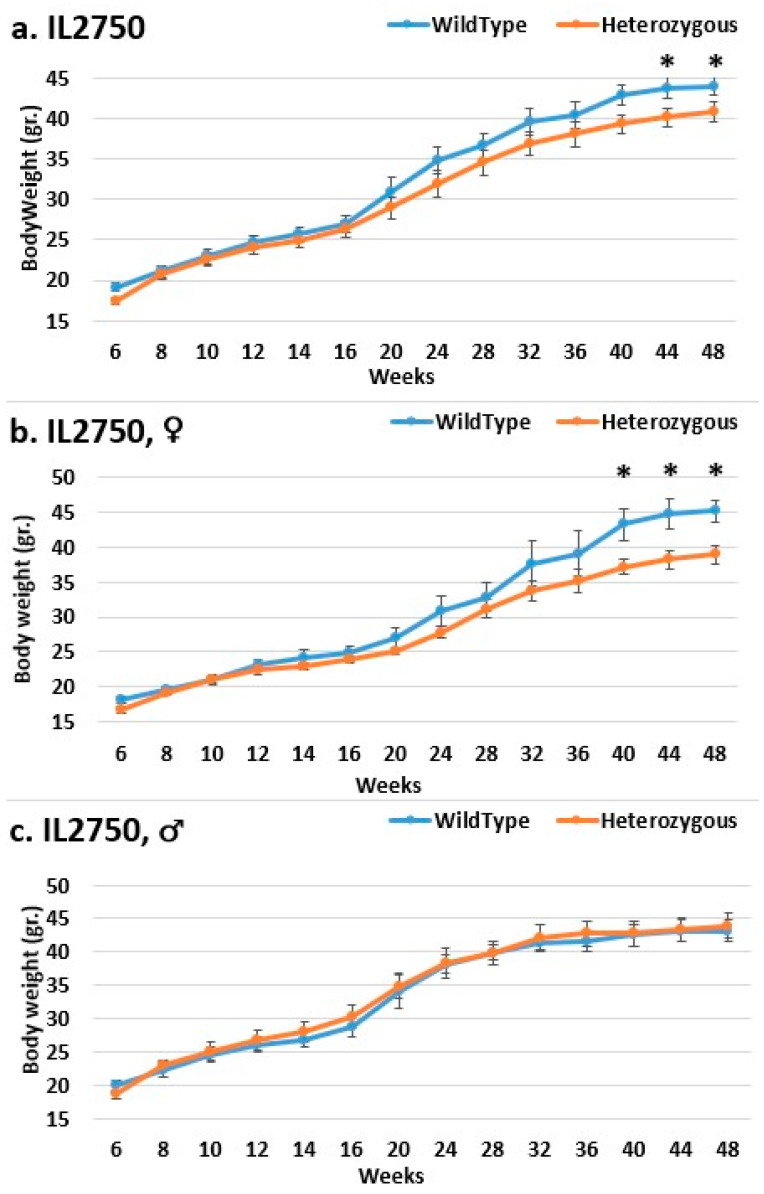
Body weight changes in wild-type and Smad4+/− mice at 14 time points for line IL2750 (6–48 weeks old). The X-axis depicts time points (in weeks), while the Y-axis depicts body weight values. From week 20 until the end of the experiment, one may observe a difference in the weight of mutant mice compared to that of control mice in both the overall (**a**) and female groups (**b**). Only from week 40 is a significant difference demonstrated. There is no difference in the male group (**c**). * Shows a significant variation between the two lines (values) at the specific time point.

**Figure 3:**
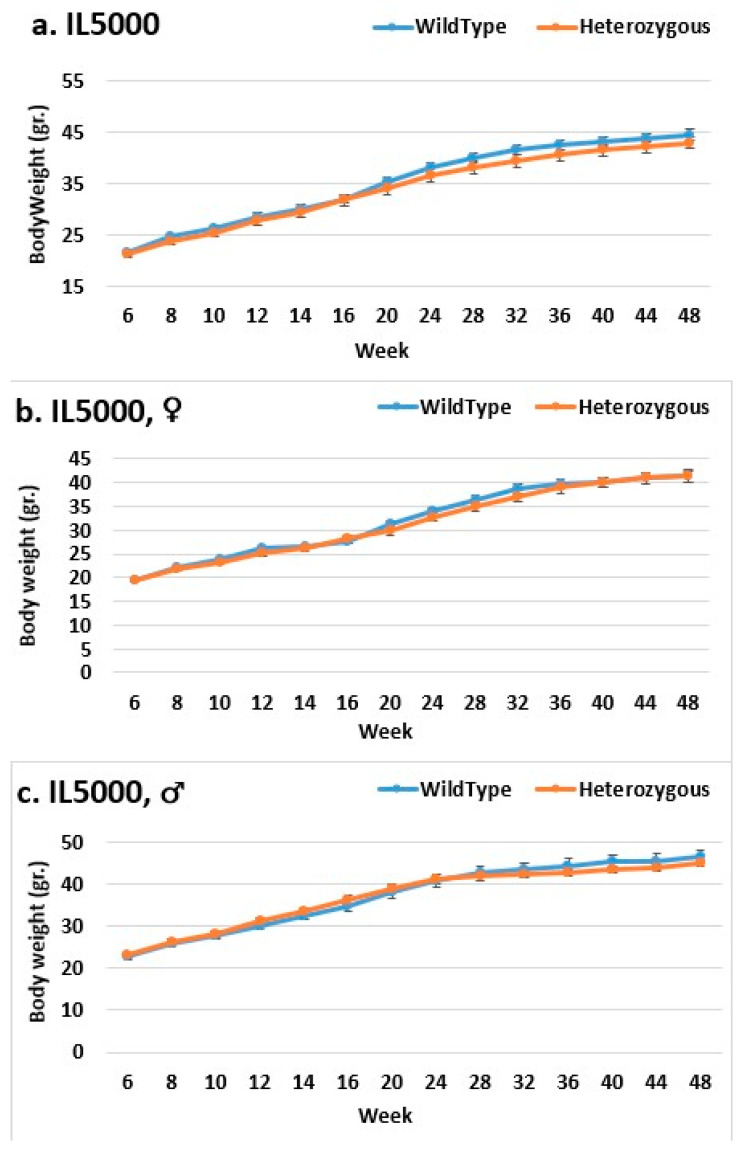
Body weight changes in wild-type and Smad4+/− mice at 14 time points for line IL5000 (6–48 weeks old). The X-axis depicts time points (in weeks), while the Y-axis depicts body weight values. Throughout the experiment, the control mice weighed more than the knockout mice, and the difference rose later in the experiment but did not achieve a statistically significant difference (**a**). There was a reversal point in the female group at week 24 where the mutant mice weighed more than the control mice and vice versa thereafter, but there was no significant difference (**b**). In the male group, control and mutant mice weighed the same from week 40 to week 48, whereas control mice weighed more before that. There was no discernible difference (**c**).

**Figure 4:**
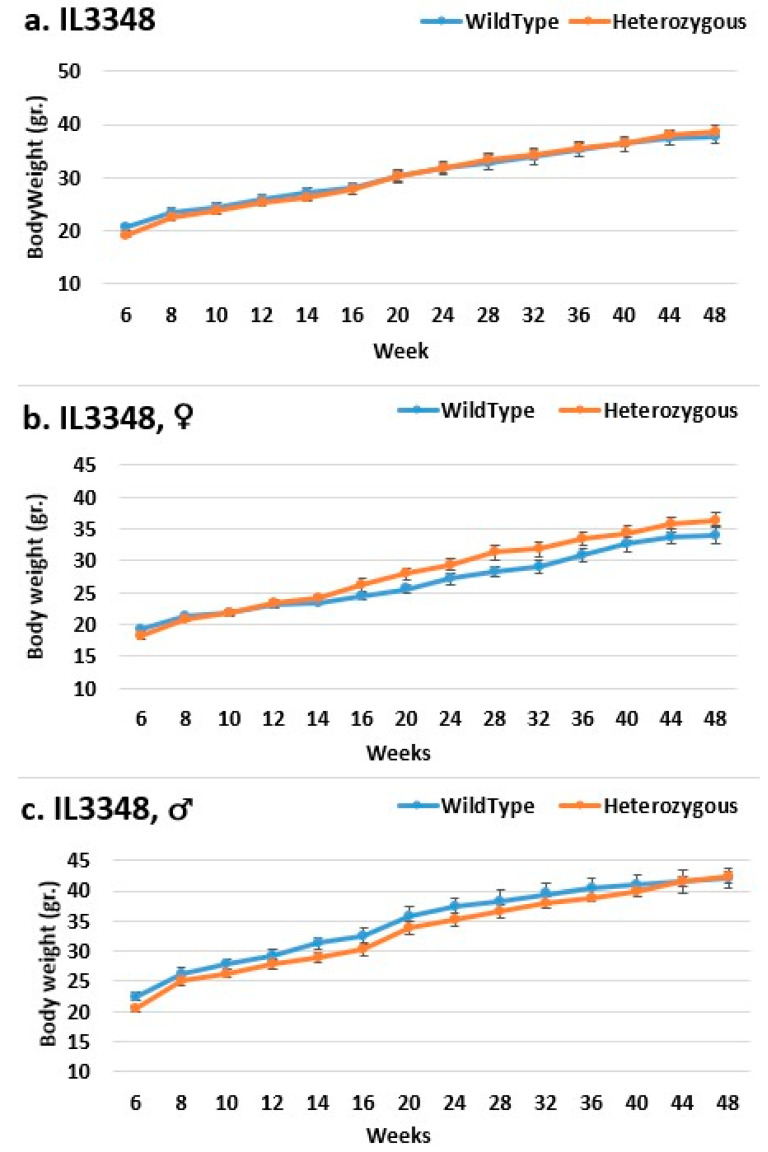
Body weight changes in wild-type and Smad4+/− mice at 14 time points for line IL3348 (6–48 weeks old). The X-axis depicts time points (in weeks), while the y-axis depicts body weight values. The mutant mice weighed about the same as the control mice throughout the experiment in the overall group (**a**). Nonetheless, when analyzed by gender, in the female group, the mutant mice weighed more than the control mice from around week 14 to the end of the experiment, but the difference was not statistically significant (**b**). In contrast, in the male group, the control mice weighed more than the mutant mice from the beginning of the experiment until week 40, but there was no significant difference, and by weeks 44 and 48, their weight was nearly identical (**c**).

In the overall and female groups, the control mice weighed significantly more than the mutant mice in line IL2750 (Figure 2). Even at the end of the experiment, the control mice in the overall group beginning in week 44 and the female group beginning in week 40 weighed significantly more than the mutant mice. In the male group, there was no difference in BW. This could mean that females were more susceptible to the mutation and that it prevented them from gaining weight, just like control mice. In line IL5000, it was found that, in all groups, the control mice were heavier than the mutant mice after a few weeks (Figure 3). This occurred in females from weeks 28 to 48, in males from weeks 10–15 and 20–36, and in the overall group throughout the experiment, but with no statistical significance. In the male group of line IL3348, the control mice weighed more than the mutant mice from the start of the experiment until week 40, but there was no significant difference, and by weeks 44 and 48, their weight was nearly identical (Figure 4). The overall and female groups had a reversal point where the control mice weighed more than the mutation mice before reversing. This occurred at week 10 in the female group and week 20 in the overall group.

**Figure 5:**
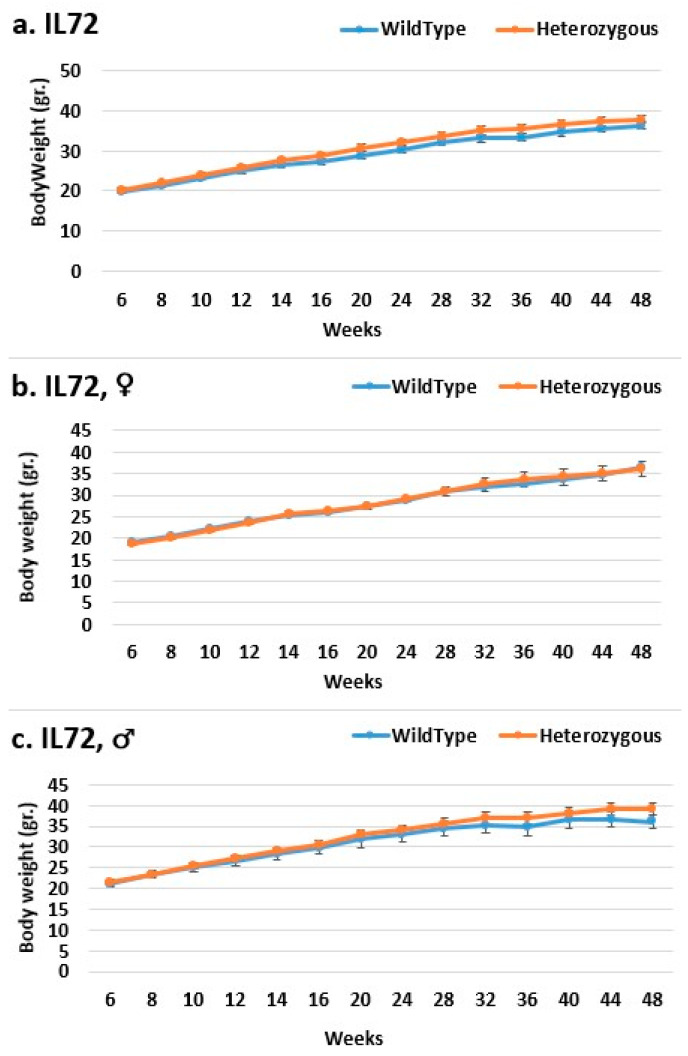
Body weight changes in wild-type and Smad4+/− mice at 14 time points for line IL72 (6–48 weeks old). The X-axis depicts time points (in weeks), while the Y-axis depicts body mass values in grams (g). We can see a difference in the weight of mice with heterozygous *SMAD4* knockout compared to control mice in the male group from week 28 until the period’s conclusion, although it is not significant (**c**). In females (**b**) and the overall group (**a**), there is no difference in BW between mutant and wild-type mice.

**Figure 6:**
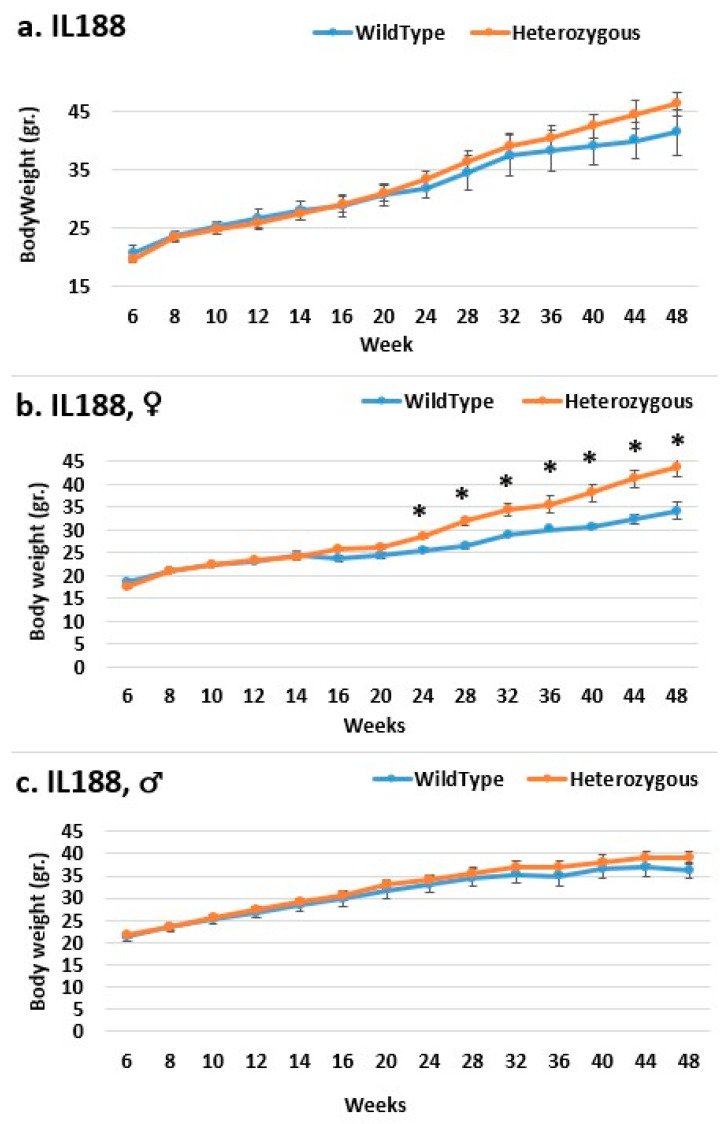
Body weight changes in the wild-type and Smad4+/− mice for line IL188 at 14time points (6–48 weeks old). The X-axis represents the time points (in weeks), while the Y-axis represents values of body mass. We can see a difference in the weight of mice with heterozygous *SMAD4*knockout compared to control mice in all groups (males (**c**), females (**b**), and overall (**a**)) from week 24 to the end of the period, although only the female group from week 24 shows a significant difference. * Shows a significant variation between the two lines (values) at the specific timepoint.

**Figure 7:**
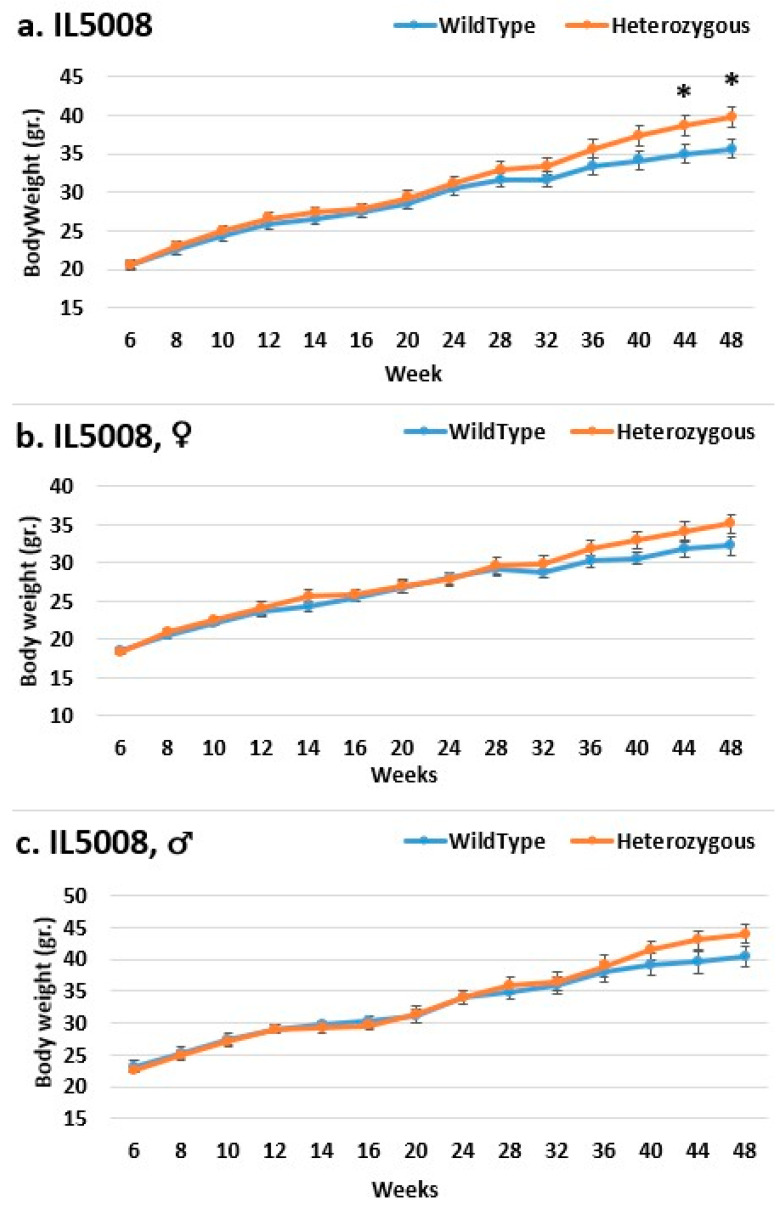
Body weight changes in wild-type and Smad4+/− mice at 14 time points for line IL5008 (6–48 weeks old). The X-axis depicts time points (in weeks), while the Y-axis depicts body weight values. In this line, mutant mice weigh more than control mice in all three groups ((**c**) males, (**b**) females, and (**a**) overall); however, the weight difference grew during the experiment and was only significant in the overall group at weeks 44 and 48. * shows a significant variation between the two lines (values) at the specific time point.

Line IL72 is the most resistant to the body weight mutation, especially in the overall and female populations (Figure 5). Throughout the experiment, the mutant mice weighed nearly the same as the control mice. Throughout the experiment, there was an increase in weight difference, favoring the mutant mice in the male group, but this was insignificant. In all groups in line IL188, the mutant mice weighed more than the control mice. Among all experimental groups, the female groups were the most affected by the mutation regarding weight (Figure 6). At the start of the experiment, line IL5008 was resistant to the effect of the mutation on weight; mice with the mutation weighed nearly as much as control mice (Figure 7). However, beginning at week 36, the weight difference between the mutant mice and the control mice in the female and male groups increased throughout the experiment but was not statistically significant. However, the difference in the overall group began earlier, at week 28, and continued to rise throughout the experiment, reaching a significant level at week 44.

**Figure 8:**
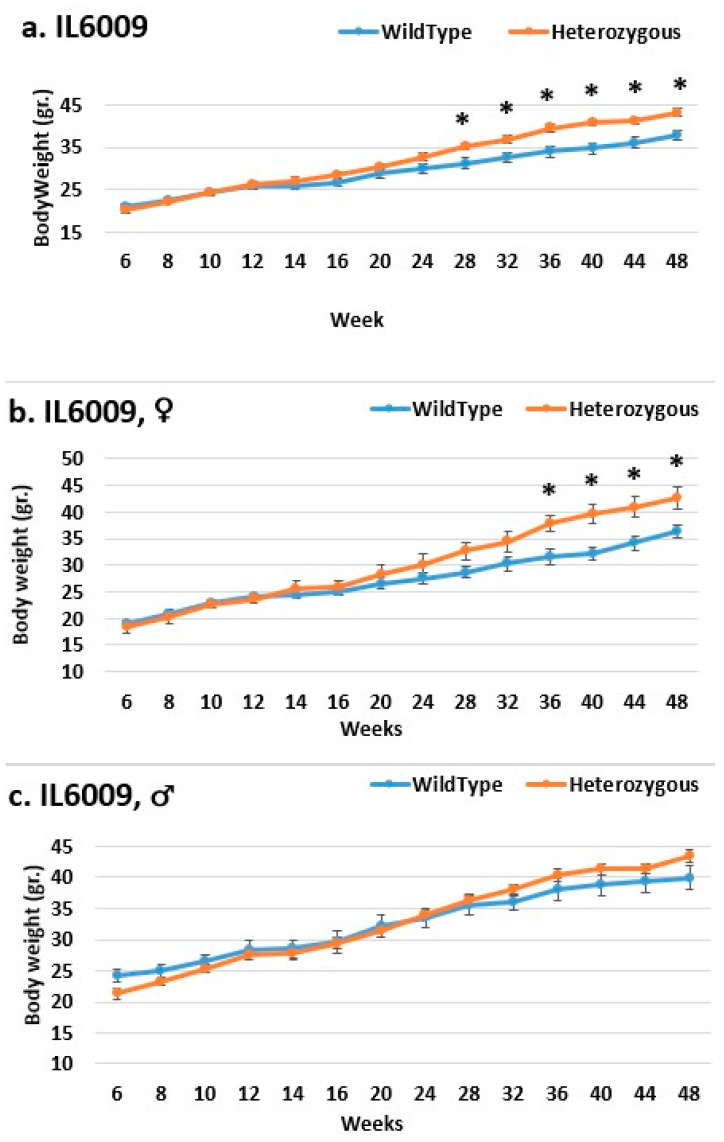
Body weight changes in wild-type and Smad4+/− mice at 14 time points for line IL6009 (6–48 weeks old). The X-axis depicts time points (in weeks), while the Y-axis depicts body weight values. From week 14 through to the end of the period, one may observe a difference in the weight of mutant mice compared to that of control mice in both the (**b**) female and (**a**) overall groups. Only from week 28 is a significant difference in the overall group demonstrated, and, starting from week 36, a significant difference is demonstrated in the female group. In the male group (**c**), there was no significant change; until week 12, the control group weighed more than the mutant. From the start through to the end of the experiment, the mutant mice weighed more than the control mice, but the difference was not significant. * Shows a significant variation between the two lines (values) at the specific time point.

**Figure 9:**
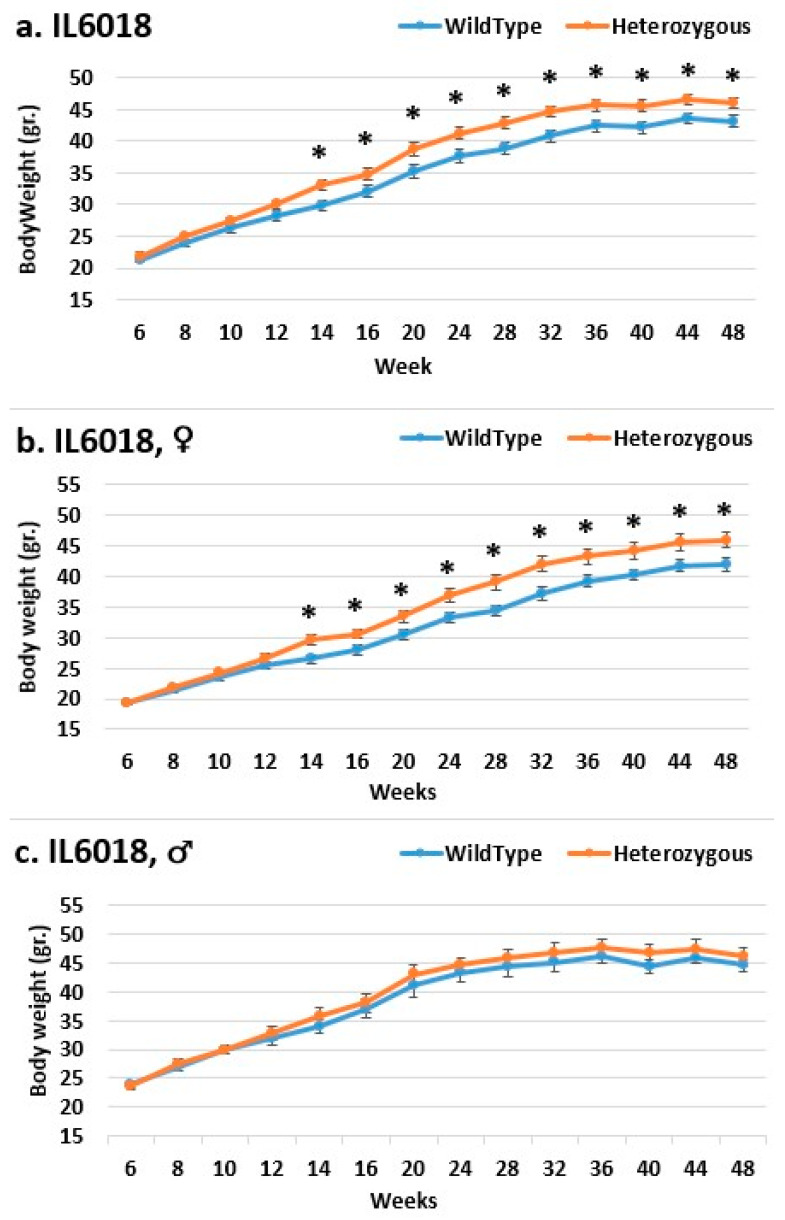
Body weight changes in wild-type and Smad4+/− mice at 14 time points for line IL6018 (6–48 weeks old). The X-axis depicts time points (in weeks), while the Y-axis depicts body weight values. One may see a substantial difference in the weight of mutant mice compared to that of control mice in both the (**b**) female and (**a**) overall groups from week 14 to the end of the experimental period. There is no difference between the (**c**) male groups. * Shows a significant variation between the two lines (values) at the specific time point.

**Figure 10:**
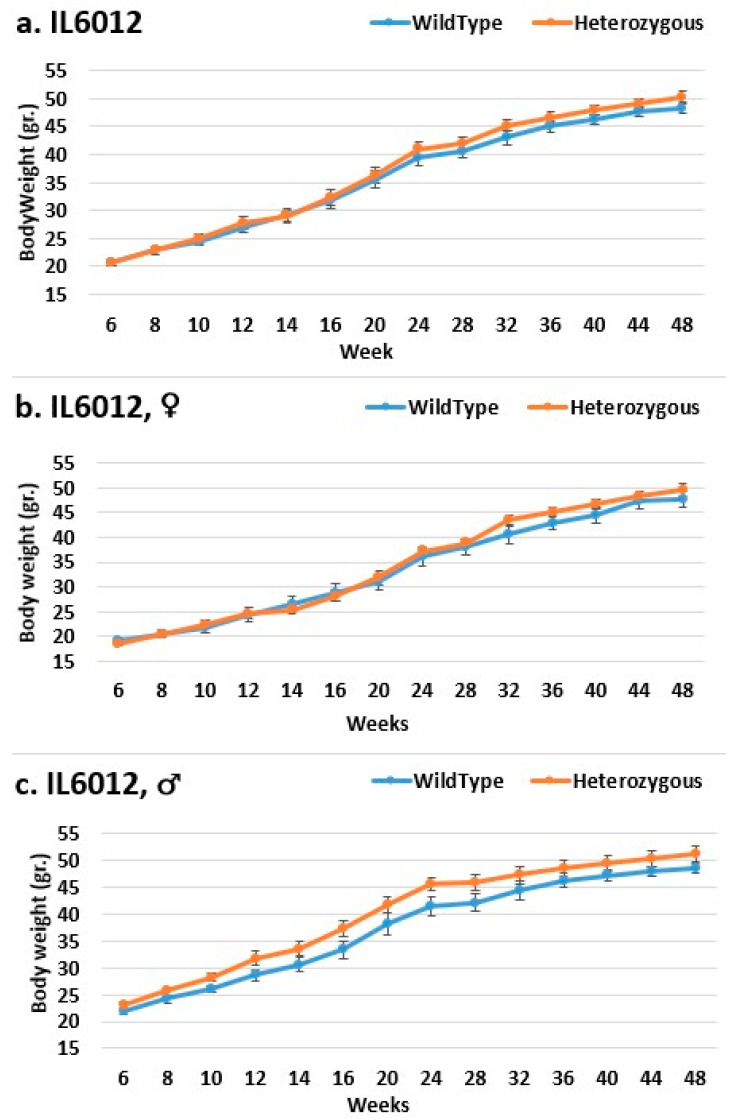
Body weight changes in wild-type and Smad4+/− mice at 14 time points for line IL6012 (6–48 weeks old). The X-axis depicts time points (in weeks), while the Y-axis depicts body weight values. In the (**a**) overall group and the (**b**) female group, the weight of the mutant mice was practically identical to that of the control mice from the beginning of the experiment until about week 20, after which the mutant mice weighed more than the control mice until the end of the experiment. In the (**c**) male group, mutant mice consistently weighed more than control mice throughout the experiment without any significant difference.

In line IL6009, from week 14 until the end of the experimental period, we observe a difference in the weight of mutant mice compared to that of control mice in both the female and overall groups. Only from week 28 is a significant difference demonstrated in the overall group, as well as from week 36 in the female group. In the male group, there was no significant difference until week 12; the control group weighed more than the mutant mice. From the start through to the end of the experiment, the mutant mice weighed more than the control mice, also without significance (Figure 8). In line IL6018, a significant difference in the weight of mutant mice compared to that of control mice in both the female and overall groups from week 14 to the end of the period was observed, but no difference was observed between the male groups (Figure 9). In contrast, the male group demonstrated a difference inline IL6012 throughout the experiment; the mutant mice were heavier than the control mice, but this difference was insignificant. In the female and overall groups throughout the experiment, the weight differences were negligible (Figure 10). 

### 2.4. Percentage of BW Gain (g) of CC Lines after 48 Weeks

**Figure 11:**
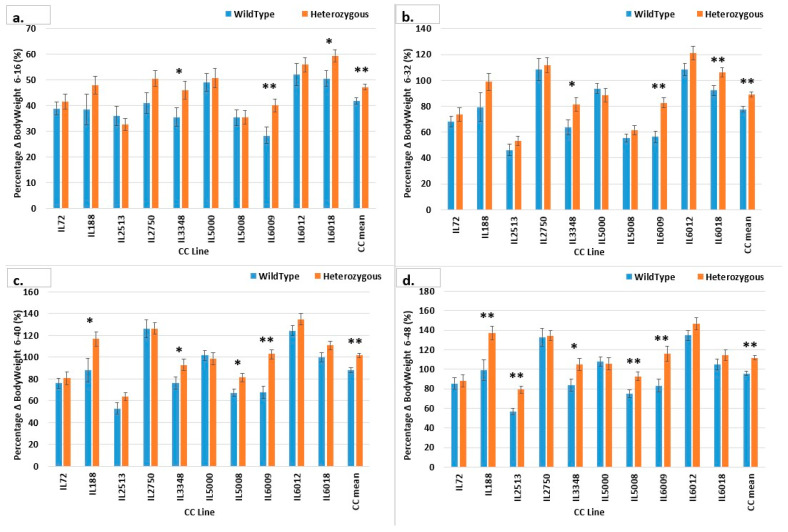
Percentage body weight gain (g) of ten different CC lines after 48 weeks of BW monitoring in the overall F1 population (males and females). (**a**) shows BW percentage gain in 10 different lines of F1 population with heterozygous *SMAD4*knockout vs. wild type until week 16; BW percentage gain until week 32 is shown in (**b**); (**c**) shows the percentage increase in BW until week 40, and (**d**) shows the percentage increase in BW until week 48. * Shows a significant variation at *p* < 0.05 between the two mutant and wild type (values), while ** *p* < 0.01.

Figure 11 shows the different %BW responses among lines, which may support our proposed study. F1 mice with the Smad4 mutation gained 47.211% more weight than wild-type F1 mice (41.753%) by week 16; subsequent body weight gains were 89.2% compared to 77.67% by week 32, 101.6% versus 87.97% by week 40, and 111.8% versus 95.69% by week 48. IL6009 and IL3348 showed a significant pattern. For lines IL5008 and IL188, the difference started to be significant only towards week 40; the same was found for IL2513 in week 48. Line IL5000 showed that the control mice gained more weight than the mutant mice from %BW 32–6 to %BW 48–6, and the same was found in line IL2513 but only in the first %BW 16–6, after which the trend reversed, and the difference grew in favor of the mutant mice for %BW 48–6. In line IL6018, the difference between mutant and control mice’s weight gain decreased and was no longer significant at %BW 40–6 and %BW 48–6. In lines IL72 and IL2750, at the start of the experiment, mutant mice gained more weight than control mice, but by the experiment’s end, average weight gain was almost identical. Figures S3–S6 shows the %BW of mutation mice versus that of control mice, with each line shown separately in addition to the total mean of all mice by gender, comparing males and females with and without the mutation. As the experiment progressed and the mice aged, the weight differences between mutant and control mice, as well as between males and females with and without the mutation, increased. Most of the lines showed a distinct pattern throughout the experiment. Line IL188 showed a significant weight difference between female mice with and without the mutation, which increased over time. Only for %BW 6–48 does line IL2513 show a significant difference in mouse weight between mutant females and controls and mutant males and controls. Line IL2750 demonstrates significant %BW differences between males with and without the mutation at weeks 6–16 and 6–32, and these differences diminished as the trial progressed. The mutation eliminated gender differences by week 48.

Line IL2750 shows significant differences in %BW only for %BW 16–6 and %BW 6–32, and these differences diminished as the experiment progressed. In those weeks, males and females with heterozygous *SMAD4* knockout gained weight differently; however, as the experiment progressed, both sexes gained weight similarly by week 48. Comparing mutant and mutation-free females In line IL3348, the difference decreases but remains significant until %BW 40–6. For %BW 16–6 and %BW 6–32, male control mice are heavier than females, and the difference decreases over time. Line IL6009 has a large difference between mutant and non-mutant males. For %BW 6–32, mutation-free and mutant females differed significantly. Male and female mutant mice had significantly different %BW 6–40. Once a difference between male and female control mice is introduced, %BW 6–48 becomes significant in all groups.

Neither male nor female IL6012 mice differed significantly between mutant and control mice. In the mutant group (6–32, 6–40, and 6–48) and the control group (%BW 48–6), females have a higher %BW than males. Line IL6018 showed a significant difference between mutant and control females early in the experiment, but only until %BW 40–6. In the mutation group and in control mice, %BW 48–6 favors females. Mutant mice started at %BW 6–32 and control mice at %BW 6–40.

Males gained %BW faster than females at the beginning of the experiment in both groups, i.e., mutant mice and control mice in the CC population, but this trend reversed over time, and by the end of the experiment, females gained significantly more weight in both groups. This is true for lines IL6000, IL6012, and IL6018 and for lines IL72 and IL5000, but it is insignificant. The %BW of line IL5008 began with females gaining more %BW than males, but this trend reversed with no significant differences. IL3348 males gained %BW more so than females, and only the control group showed a significant difference for %BW 16–6 and %BW 32–6. Line IL2513 begins with a large difference in %BW between males and females and ends with a dramatic decrease.

### 2.5. Computational Methods

#### 2.5.1. Heritability

This study aimed to discover whether BW phenotypic variance has a genetic basis in Smad4knockout F1 populations. Table 3 summarizes the heritability (H2) values calculated to answer this question. One-way ANOVA was used to calculate the heritability of sex- and genotype-specific characteristics. The different traits calculated are as follows: %BW 6–16, %BW 6–32, %BW 6–40, and %BW 6–48 for both sexes and genotypes. In both control and mutant groups, female %BW had a higher H2 value than males. Wild-type sexes have higher heritability than mutants. In the fourth group, %BW 6–16 values were low and leaned toward 0. Both genotype groups had the same H2 for%BW 6–32 (0.52). In females of both genotypes, H2 in %BW 6–32 is highest (0.62 and 0.64, respectively).

#### 2.5.2. Regression Models

To predict the genotype (0/1) of a mouse based on its sex and 14 recorded weights, Table 4 displays the results of six distinct regression algorithms for each line. Most lines in this table have low AUC values. The logistic regression model predicted the genotype of four lines (IL188, IL2513, IL2750, and IL6009), making it the most effective. Line IL2513, using logistic regression, had the greatest AUC (0.875). IL6009 has multiple high values, including SVC = 0.836, LR = 0.82, NaBa = 0.787, and KNN = 0.798. No model was able to predict the IL3348, IL5000, IL5008, and IL6012 lines with scores greater than 0.6.

#### 2.5.3. Model Details

We predicted the BW of mice between weeks 8 and 48. The model was evaluated by means of fivefold cross-validation. The mean scores are presented per line in Table 5. Lines IL6018, IL5008, IL2513, and IL3348 are the most predictable in terms of mouse weight, with line 5008 being the most predictable. Beginning in week 6, line IL2570 is highly predictable. Lines IL6009 and IL188 are the least predictable, while line IL72’s results improved with more data but remained unpredictable.

#### 2.5.4. Correlation Analysis between the Studied Traits

**Figure 12:**
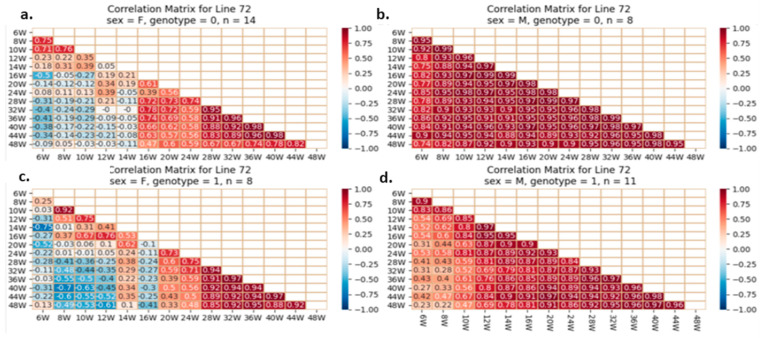
Heatmap and Pearson correlation between the body weight of female and male mice of line IL72, wild-type or mutant, between various weeks. (**a**) wild-type female, (**b**) wild-type male, (**c**) mutant female, (**d**) mutant male. The r values ranged from minimum (−1) to maximum (1).

**Figure 13:**
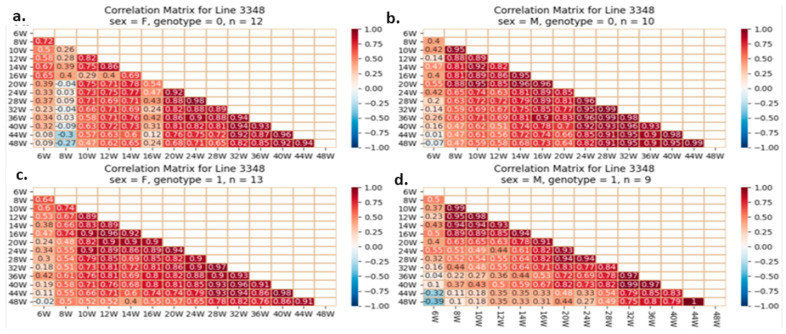
Heatmap and Pearson correlation between the body weight of female and male mice of line IL3348, wild-type or mutant, between various weeks. (**a**) wild-type female, (**b**) wild-type male, (**c**) mutant female, (**d**) mutant male. The r values ranged from minimum (−1) to maximum (1).

**Figure 14:**
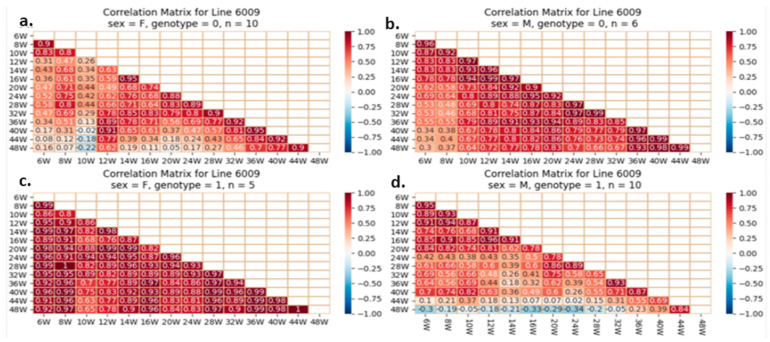
Heatmap and Pearson correlation between the body weight of female and male mice of line IL6009, wild-type or mutant, between various weeks. (**a**) wild-type female, (**b**) wild-type male, (**c**) mutant female, (**d**) mutant male. The r values ranged from minimum (−1) to maximum (1).

One of our proposed experiments was to evaluate if initial and BW gain correlate with adult weight in mice with or without the studied mutation. Pearson’s correlations quantify BW correlations and direction (increase or decrease). A value of +1 indicates a perfect positive relationship and correlation, −1 indicates a perfect negative one, and 0 indicates no relationship. Early and late body weights were correlated using heatmaps. Each line reflects mice’s weekly body weights. The fourth groups on each line are separate. Examples of heatmaps for lines IL72, IL3348, and IL6009 are shown below. The variations observed between different CC lines and within the same line are presented in Figure 12, Figure 13 and Figure 14.

In later weeks, the weight of mice with a positive and stronger correlation tends toward 1; this correlation begins at week 28 and strengthens by week 44; however, this correlation is poorly investigated in mutant males of line IL6009 and is weaker in control females. Line IL72 shows a color difference between the four groups’ initial and advanced weights. The relationship between initial weight and the final week’s weight is strong and positive in male controls but weaker in mutants. Mutant females have a worse relationship than normal females. Line IL6009’s mutant females show a strong and positive relationship throughout the experiment, beginning with week 6 weights, being correlated strongly with all other weeks. Week 10’s correlation is the lowest. The male control shows a strong positive correlation throughout the experiment. Week 10 weakens and strengthens it. Males with heterozygous *SMAD4 knockout* have a negative correlation between final weight at week 48 and weight from weeks 6 to 32.

## 3. Discussion

Although BW is a reliable, valid, and easy-to-measure metric, its short-term dynamics are poorly understood because it is difficult and time-consuming to make frequent longitudinal measurements of BW, and previous studies estimating BW typically used infrequent measurements (e.g., every 6–12 months). A few studies have examined weekly or seasonal patterns in BW, but no study, to our knowledge, has estimated BW over the long term in the setting of a tumor suppressor knockout mice model. Our results showed that BW varies significantly between CC strains and within lines depending on whether Smad4 is knocked out, demonstrating that CC mice comprise an excellent platform for studying BW interactions and genotypes. Additional lines with and without the mutation must be weighed for phenotype–genotype association analyses. Once acquired, such information can forecast weight gain risk and build genetically based preventative and treatment techniques.

Animal welfare is routinely assessed by monitoring BW [38]. Typically, a loss of 20 percent of BW is used as the default endpoint [39]. However, these can be difficult to determine precisely in cancer models since an increase in tumor mass can mask the loss of overall BW [40]. Unexpected weight loss is frequently one of the earliest detectable signs of cancer. As the disease progresses, wasting and muscle loss, also known as cachexia [41], can worsen and contribute to death. On the other side, overweight-related cancers are becoming more common, making this disease spectrum a public health priority. Obesity is a primary predictor of cancer’s rising incidence and prevalence, and it may soon overtake smoking as the leading avoidable cause of cancer [6].

Heatmaps emphasize the differences between lines. There were different correlations between initial BW and final BW between lines and within lines regarding sex, and this was exacerbated in the presence of mutant tumor suppressors. Weight gain differs in populations with different genetic backgrounds, as observed previously by Lone et al. [40]. Some lines gained more weight in the presence of heterozygous Smad4 knockout, while others gained less. It also depended on the gender of the mice, but, in general, the presence of the mutation resulted in overweight mice. The finding that null alleles of mouse genes frequently reduce but occasionally increase BW is not unique [42]. To our knowledge, no previous research has examined the overall effect of gene deletion on mice’s BW because knockout studies are often conducted to answer specific gene research questions. However, Daniel et al. scanned the Mouse Genome Database for each knockout gene on six chromosomes to objectively analyze the effect of gene knockouts on BW [43]. They estimated that more than 6000 genes contribute to mouse body size, and 30% of null alleles result in a mouse with lower BW, while 3% result in a mouse with higher BW [43]. The genome favors weight gain with ten times as many genes increasing size as those decreasing. This supports the theory that mice are “hard-wired” to favor positive energy balance [44,45].

In this research, we carried out a comparative analysis of various ML algorithms for predicting the genotype of mice. It was shown that the ML algorithm logistic regression provided better results than common regression algorithms for prediction. A successful classification of a genotype-based solely on weight has undeniable clinical utility. Although the cost of sequencing has decreased significantly over time, it is still not a common practice. Line IL6009 exhibited the highest level of predictability, with an AUC > 0.75 predicted by four different models. As demonstrated by the correlation matrices, this line’s behavior differed between the two genotypes. In line IL6012, for instance, the heatmaps are extremely similar, and the classification results are also lower. The results clearly show differences between lines. In other words, an individual’s entire genotype influences the predictability of this heterozygous Smad4 knockout gene. The interactions of this protein with other proteins within the cell could be a possible explanation for this phenomenon.

To predict the BW of mice, global outcomes are anticipated; the more information we provide to the algorithm, the more accurately it predicts. However, even when we only provide the algorithm with data from week 8, the results are quite good. The line-separated results are quite unexpected. While some lines, such as IL5008 and IL2513, are highly predictable from week 8 or even week 6, others, such as IL188, are unpredictable. In addition, we observe that adding more data to the algorithm hinders its performance for some lines, including lines IL5000 and IL6009. The outcomes vary between lines. We believe these variations are due to the different genotypes of the lines, as the behavior varies based on genotype. Some behaviors are more predictable than others, particularly when a linear regression model is used. From what we have seen, for most lines, it seems like there are two linear behaviors with a specific changing point slope-wise. In the future, it could be interesting to try to predict the time of change in the slope based on the initial features alone. Studies in mouse strains with various genetic backgrounds and established genetic structures provide unparalleled opportunities to find and analyze trait-related genomic loci. Additional lines with and without mutations must be weighed for phenotype–genotype association analyses. Once acquired, such information can forecast weight gain risk and build genetically based preventative and treatment techniques.

## 4. Materials and Methods

### 4.1. Ethical Aspects of the Project

It is important to note that all animal experiments in this study were compliant with national standards for the care and use of laboratory animals, and the experiment was reviewed and approved by Tel Aviv University’s Institutional Animal Care and Use Committee (IACUC), with an approved number (01–19–044). Mice were monitored daily for their overall health status. Mice that showed losses of around 10% of their BW between two measure points, or 20% overall of their initial body weight, or those that were observed to be suffering (less movement and activity) based on consultation with the veterinarian at the small animal unit, were terminated. 

### 4.2. Study Cohort

F1 (Smad4+/−x CC) and control F1 (Smad4+/+x CC) mice were generated by crossing male mice of C57Bl/6J carrying Smad4+/− with 10 different CC females; the mice were expected to show significant differences in their susceptibility to spontaneous cancer development as described previously in a perspective review [46]. The CC mice were supplied by the animal facility of Tel Aviv University and raised in the facility. They were maintained under ethical standards of humidity and temperature (21–23 °C).

### 4.3. Study Design

F1 mice were weaned at 4 weeks of age and maintained until 48 weeks of age on a standard rodent chow diet (TD.2018SC, 18% Kcal from fat, 24% from protein, and 58% from carbs; Teklad Global, Harlan Inc., Madison, WI, USA) with a maximum of five mice per cage. The experiment began when the mice were four weeks old. Throughout the trial, BW was determined biweekly until week 16 and then monthly until week 48. Throughout the trial, mice had free access to food and water.

### 4.4. Genotype

At 4 weeks old after weaning, 0.5 cm tail biopsies were collected from all F1 crossed (Smad4+/− x CC) mice and DNA was extracted using NaOH boiling protocol [47]. Using the protocol provided by Jackson Laboratory(Bar Harbor, ME, USA), the vendor providing the C57BL/6J- Smad4+/− mice were genotyped using polymerase chain reaction (PCR) protocol to distinguish the Smad4/− mutant allele from the wild type (Smad4+) using the following primers: ID# 30403 (TGT AGT TCT GTC TTT CCT TCC TG), ID# 30404 (ACT GAC CTT TAT ATA CGC GCT TG), and ID# oIMR2088 (AGA CTG CCT TGG GAA AAG CG), which were designed by Jackson Laboratory. For Smad4 wild-type alleles, we used the primers 30403 and 30404, while for the mutant allele, we used 30404 and oIMR2088 primers. For later identification, each mouse was labeled by ear cuts.

### 4.5. Heritability and Genetic Coefficient Variation

Heritability quantifies the proportion of phenotype variation attributable to genetic variation. Here, we calculated the broad-sense heritability using the ANOVA data using the formula below:

(1) H2=Vg/(Vg+Ve)

*H2* is the heritability, *Vg* is the genetic variance between the *CC* lines, and *Ve* is the environmental variance. Considering the heritability results, we calculated the genetic coefficient of variation.

### 4.6. Computational Methods

#### 4.6.1. Classification Models

Classification is a data mining (machine learning) method to predict group connectivity for data instances. Decision tree (DT), Random Forest (RF), Naïve Bayes, K-Neighbors, Support Vector Machine Classifier (SVC), and logistic regression (LR) classifiers were implemented for further analysis. They were all applied using K-fold cross-validation (K = 4) implementation of the Python package Scikit-Learn (https://scikit-learn.org/stable/, accessed on 21 September 2023). All models were assessed by the average AUC of the ROC curve, using a 4-fold cross-validation 3 times for each algorithm.

#### 4.6.2. Decision Tree

Decision tree algorithms are the most used algorithms in classification models. DT algorithm constitutes a convenient modeling technique and simplifies the classification process. The decision tree is a transparent mechanism and assists users in following a tree structure easily to see how a decision is made. In this study, we used Scikit-Learn’s default decision tree implementation with a maximum depth of 10.

#### 4.6.3. K-Neighbors

The K-nearest neighbor (KNN) technique is measured with respect to the value of k, which defines how many nearest neighbors need to be examined to describe the class of a sample data point. In this study, we used Scikit-Learn’s default implementation for its K-Neighbors classifier (5 neighbors).

#### 4.6.4. Random Forest

RF is a classification technique that uses many decision trees. RF is a multifunctional machine-learning technique. It can perform the tasks of both prediction and regression. Additionally, RF is based on bagging, and it plays an important role in ensemble ML [48]. RF, thus, has been implemented in biomedicine research vastly [49]. In this study, we used Scikit-Learn’s default implementation for random forest with 100 trees.

#### 4.6.5. Naïve Bayes

A Bayesian Network (BN) refers to a graphical model used for probability associations between a set of variables. In this study, we used Scikit-Learn’s default implementation for Naïve Bayes (Gaussian).

#### 4.6.6. Support Vector Machine Classifier

SVM is a supervised learning method that examines data and sorts them into two categories. An SVM outputs a map of the sorted data with the margins between the two categories as far apart as possible. SVMs are used in text categorization, image classification, handwriting recognition, and the sciences.

#### 4.6.7. Logistic Regression (LR)

Logistic regression is a statistical analysis method used to predict a binary outcome, such as yes or no, based on prior observations of a data set. A logistic regression model predicts a dependent data variable by analyzing the relationship between one or more existing independent variables.

#### 4.6.8. Model Details

We used a chained linear regression algorithm in this research. This algorithm’s basic idea is to predict a set of ordered features using the basic features and previously predicted features. We used the following algorithm to predict mice’s body weight from week 8 to week 48 in our study:

$$w_{1}=last\;week\;we\;let\;the\;algorithm\;know\;its\;data\;1.\;Predict\;w_{2}\;using\;Sex,\;Genotype,\;BW6,\;\ldots ,\;w_{1}\;2.\;For\;every\;week,\;w_{k},\;from\;w_{2}\to 48\;\left(if\;data\;exists\right):\;Predict\;w_{k}\;using\;Sex,\;Genotype,\;BW6,\ldots .,w_{k-1}\;Finally,\;return\;the\;R^{2}\;of\;the\;predicted\;features\;and\;the\;actual\;features.$$

The model was assessed by 5-fold cross-validation.

## 5. Conclusions

Overweight and obesity not only increase the risk of CVD and T2D, but they also increase the risk of many different types of cancers [50]. Even though it is known that BW is a relevant variable, efforts to stop the obesity epidemic have failed [51]. The global burden of cancer has increased in tandem with the prevalence of obesity, and 13 types of cancer have been linked to fat [52]. Cancers of the breast (postmenopausal), colon–rectum, endometrium, ovary, pancreas, kidney, gallbladder, gastric cardia, liver, esophagus (adenocarcinoma), meningioma, thyroid, and multiple myeloma are cancers associated with overweight or obesity. However, there is a prominent research gap between weight gain and certain cancers linked to obesity. Consequently, there is a pressing need to monitor mice and their weight at older ages and to determine if there is a correlation between the percentage of weight gain in mutant mice compared to control groups and cancer susceptibility of various types.

In the future, it would be interesting to use the advantages of heterozygous Smad4 knockout CC mice to investigate additional phenotypes of MetS, such as insulin resistance, diabetes, and lipid metabolism, in addition to cancer susceptibility. Understanding how genetic background affects metabolic profiles in a heterozygous Smad4 knockout setting is essential for precision medicine and individualized pharmaceutical therapies. Our research with CC strains and mutant mouse models demonstrates that genetic background plays a pivotal role in determining an individual’s response to weight gain and cancer susceptibility. This finding underscores the need for personalized and precision medicine approaches. Other researchers can leverage our insights to design experiments with specific genetic backgrounds to tailor treatments and interventions to individual patients. By understanding how genetics influence metabolic profiles and disease susceptibility, we can work towards developing targeted therapies for various conditions, including obesity-related cancers. The CC mouse strains studied have proven to be an invaluable resource for understanding the interactions between genetics and BW dynamics. Researchers in genetics, cancer biology, and metabolic disorders can build upon our work to investigate a wide range of phenotypes related to metabolic syndrome, including insulin resistance, diabetes, and lipid metabolism. This expanded research can contribute to a more comprehensive understanding of the complex interplay between genetics, environmental factors, and disease susceptibility. Our findings open the door to a new era of risk assessment and disease prevention. The knowledge gained from our study can be used to develop predictive models for assessing an individual’s susceptibility to weight gain and associated health risks. These models can be employed to identify individuals at higher risk and implement early interventions and lifestyle modifications to reduce their risk of obesity-related diseases.

## Figures and Tables

**Table 1:** Summary table of the total number (N) of F1 mice from each CC line—males and females separately assessed in each study group (wildtype vs. mutant).

	Male		Female		
	Wild Type	Mutant	Wild Type	Mutant	
IL72	8	11	14	8	41
IL188	5	8	3	8	24
IL2513	8	3	4	12	27
IL2750	6	7	6	11	30
IL3348	10	9	12	13	44
IL5000	14	10	9	12	45
IL5008	9	12	13	11	45
IL6009	6	10	10	5	31
IL6012	10	9	6	11	36
IL6018	16	18	19	15	68
				**Total**	**391**

**Table 2:** Comparing the prevalence of wild-type mice to that of mutant mice regarding the SMAD4 gene and the predominance of female mice compared to that of male mice.

	Frequency	Percent (%)	Total
**Wild Type**	188	48.1	
**Mutant**	203	51.9	
**Female**	202	51.7	
**Male**	189	48.3	
			391 (100%)

**Table 3:** Results of calculating heritability (H2) values. The heritability was calculated using one-way ANOVA for %BW 6–16, %BW 6–32, %BW 6–40, and %BW 6–48 traits that were calculated separately by sex and genotype.

		Trait	df between	df within	n	MS between	MS within	VG	H2
Female	Wild	%∆BW 16-6	10	87	8	582.107	170.343	51.47049	0.232044
		%∆BW 32-6	10	87	8	5380.415	464.75	614.4581	0.56936
		%∆BW 40-6	10	87	8	7035.673	457.407	822.2833	0.642564
		%∆BW 48-6	10	86	7.909	6986.655	612.58	792.0095	0.659886
	Mutant	%∆BW 16-6	10	97	8.909	682.415	154.546	59.2506	0.277135
		%∆BW 32-6	10	97	8.909	7073.207	497.555	738.0834	0.59733
		%∆BW 40-6	10	97	8.909	7974.515	570.821	831.0269	0.592808
		%∆BW 48-6	10	97	8.909	8705.475	614.002	908.2266	0.596643
Male	Wild	%∆BW 16-6	10	83	7.636	923.507	289.006	83.08942	0.223301
		%∆BW 32-6	10	83	7.636	3753.41	400.828	439.0286	0.522742
		%∆BW 40-6	10	83	7.636	3558.534	476.411	403.6113	0.458638
		%∆BW 48-6	10	83	7.636	4110.591	541.017	467.4442	0.463522
	Mutant	%∆BW 16-6	10	88	8.091	1230.387	201.046	127.2219	0.387555
		%∆BW 32-6	10	88	8.091	3524.053	361.562	390.8697	0.519475
		%∆BW 40-6	10	88	8.091	2554.555	403.156	265.9032	0.397429
		%∆BW 48-6	10	88	8.091	2597.145	536.887	254.6386	0.321706

**Table 4:** Results of chained linear regression algorithm to predict the latter part of body weight trajectory of a mouse based on genotype (0/1), sex, and body weight data up to the mentioned week.

Line	IL72	IL188	IL2513	IL2750	IL3348	IL5000	IL5008	IL6009	IL6012	IL6018
N	41	24	27	29	44	45	45	31	36	68
DT	0.504	0.5	0.652	0.513	0.41	0.497	0.543	0.58	0.494	0.433
NaBa	0.603	0.459	0.67	0.58	0.553	0.491	0.584	0.798	0.528	0.644
KNN	0.506	0.5	0.718	0.637	0.555	0.42	0.556	0.787	0.483	0.539
RF	0.543	0.619	0.747	0.546	0.571	0.512	0.457	0.718	0.518	0.561
SVC	0.418	0.438	0.682	0.386	0.407	0.472	0.325	0.836	0.459	0.576
LR	0.332	0.747	0.875	0.752	0.531	0.392	0.557	0.82	0.405	0.586

**Table 5:** Results of BW prediction algorithms for each line to predict the genotype (0/1) of a mouse based on its sex and 14 recorded weights.

Line	IL72	IL188	IL2513	IL2750	IL3348	IL5000	IL5008	IL6009	IL6012	IL6018
N	41	24	27	29	44	45	45	31	36	68
6W	0.29	−0.30	0.64	0.08	0.30	0.35	0.38	0.23	0.33	0.44
8W	0.24	−0.21	0.63	0.13	0.37	0.37	0.56	0.15	0.27	0.53
10W	0.26	−0.03	0.60	0.28	0.57	0.39	0.53	0.08	0.37	0.51
12W	0.35	−0.20	0.64	0.44	0.52	0.32	0.52	0.13	0.27	0.48
14W	0.43	−0.32	0.59	0.41	0.54	0.25	0.54	0.03	0.32	0.61
16W	0.32	−0.40	0.56	0.33	0.53	0.21	0.58	−0.15	0.31	0.64
20W	0.47	−1.23	0.42	0.13	0.51	0.20	0.71	−0.12	0.25	0.61
24W	0.46	−1.58	0.55	0.15	0.66	0.06	0.74	−0.28	0.40	0.61

## Data Availability

Data are contained within the article.

## Supplementary Materials

The supporting information can be downloaded at: https://www.mdpi.com/article/10.3390/ijms242216136/s1.
