# Supplementary material for: Host Genetic Background Effect on Body Weight Changes Influenced by Heterozygous Smad4 Knockout Using Collaborative Cross Mouse Population
Source: Int J Mol Sci. 2023 Nov 9;24(22):16136. doi: 10.3390/ijms242216136 (PMC10671513; doi:10.3390/ijms242216136)
Supplement: Supplementary file 1 [file ijms-24-16136-s001.zip › ijms-2652835-supplementary.pdf]

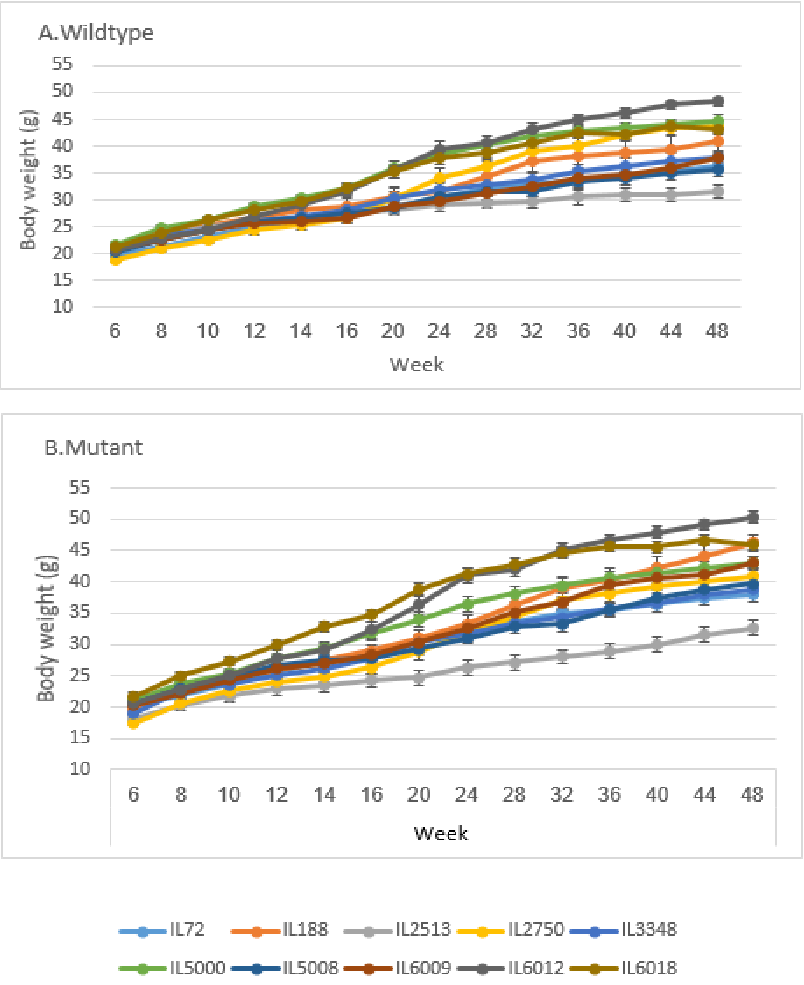

Figure S1. BW (±SE) A of the control and B Smad4+/- groups at 14 time points (8, 10, 12, 14, 16, 20, 24, 28, 32, 36, 40, 44 and 48 weeks old) of 10 different sets of 10 different CC lines. The X-axis represents the time points (in weeks) while the Y-axis represents values of body weight (g). One-way ANOVA performed for statistical analysis, $p < 0.05$.

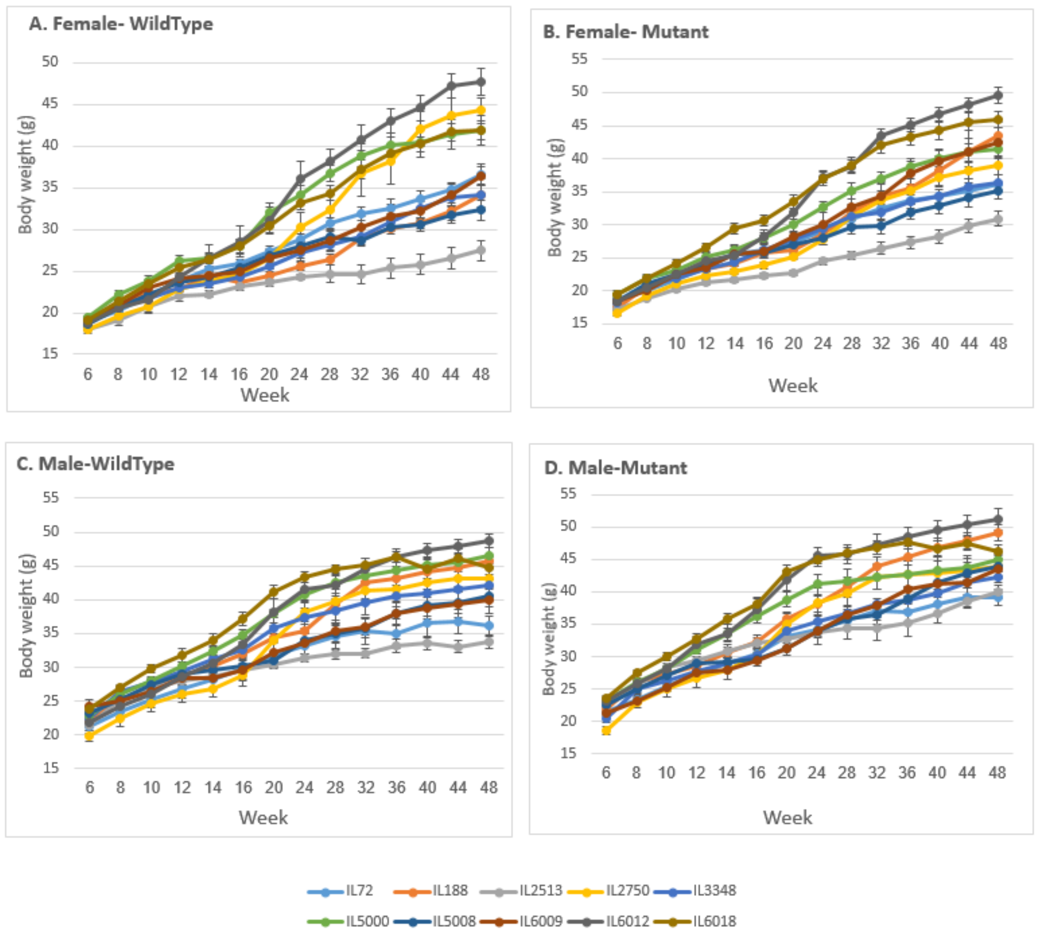

Figure S2. Body weight (±SE) of the A, C control and B, D Smad4+/- groups at 14 time points (8, 10, 12, 14, 16, 20, 24, 28, 32, 36, 40, 44 and 48 weeks old) of 10 different set of CC lines. The X-axis represents the time points (in weeks) while the Y-axis represents values of body mass (g). One-way ANOVA performed for statistical analysis, $p < 0.05$.

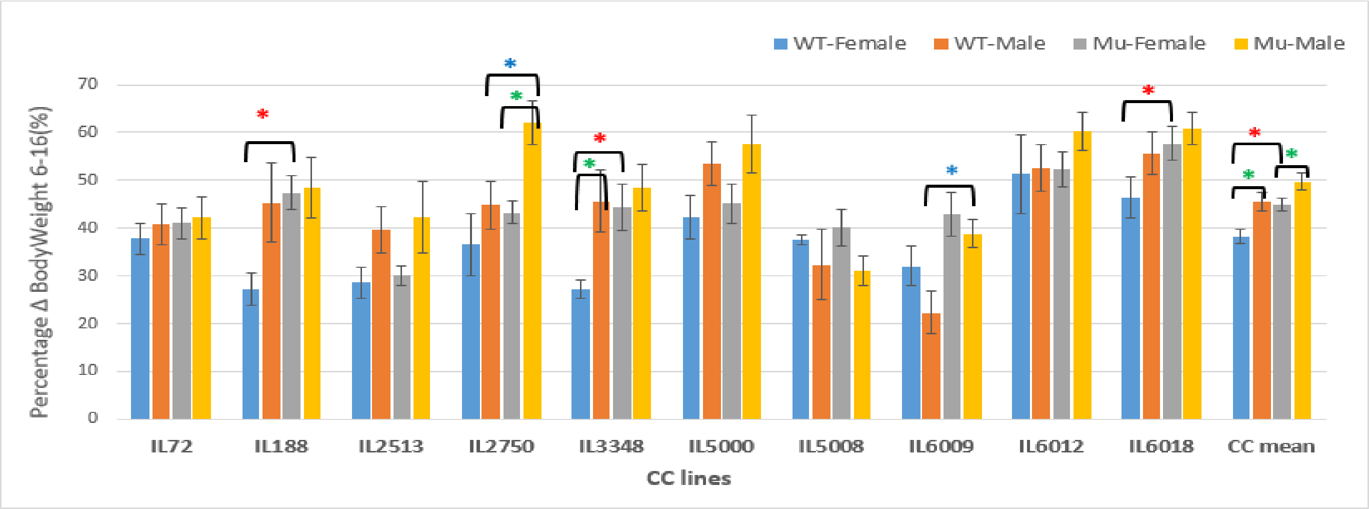

Figure S3. Percentage changes in Body weight in grams between weeks 6-16 of ten CC lines, separately for females and males, having wildtype and mutant genotypes. The X-axis depicts the various CC lines, while the Y-axis depicts the percentage change in BW over weeks 6-16.

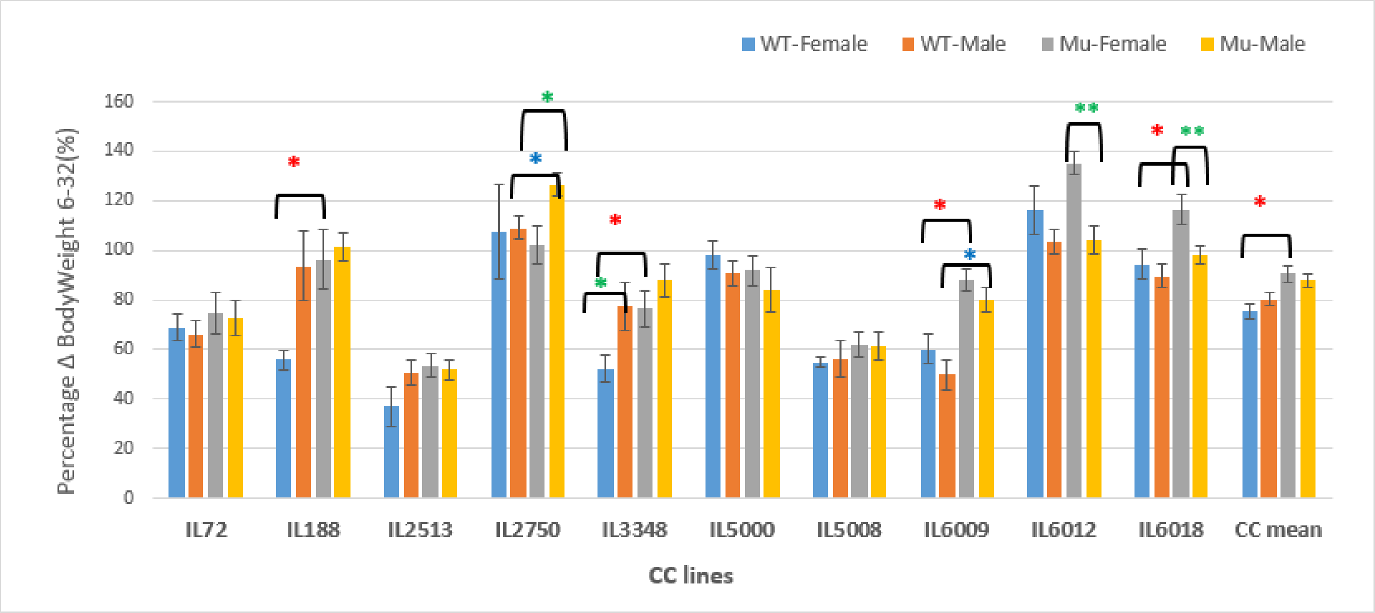

Figure S4. Percentage changes in body weight in grams between weeks 6-32 of ten CC lines, separately for females and males, having wildtype and mutant genotypes. The X-axis depicts the various CC lines, while the Y-axis depicts the percentage change in BW over weeks 6-32.

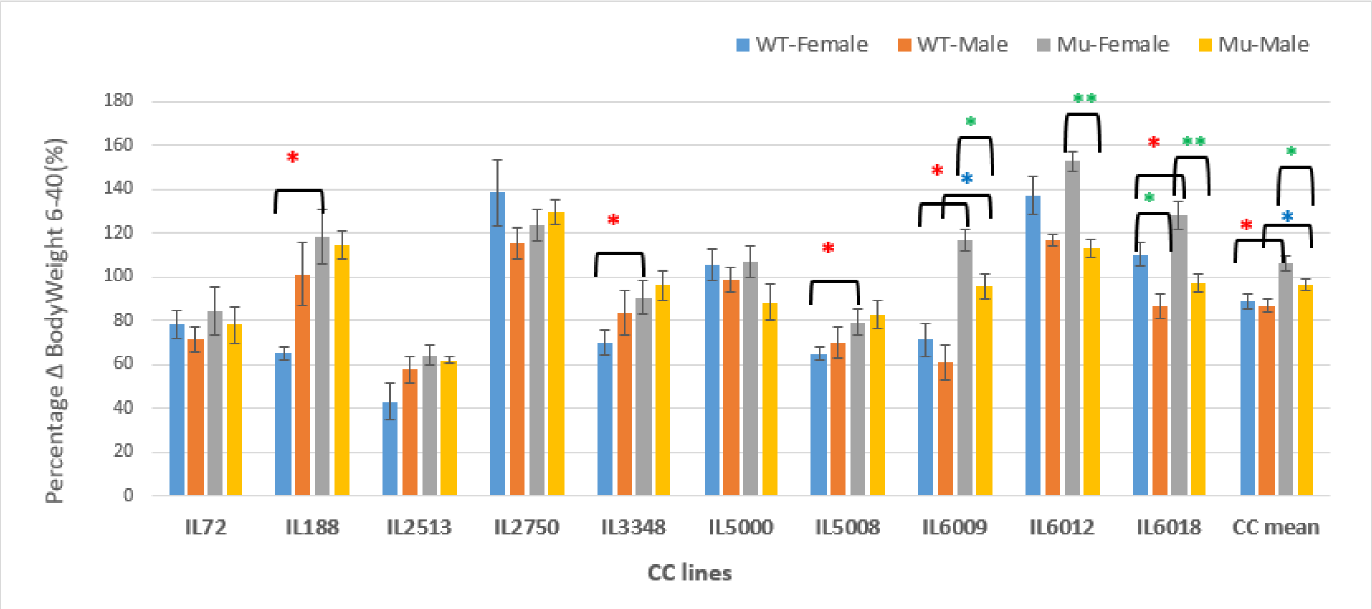

Figure S5. Changes in BW as a percentage in grams between weeks 6-40 of ten CC lines, separately for females and males, having wildtype and mutant genotypes. The X-axis depicts the various CC lines, while the Y-axis depicts the percentage change in BW over weeks 6-40.

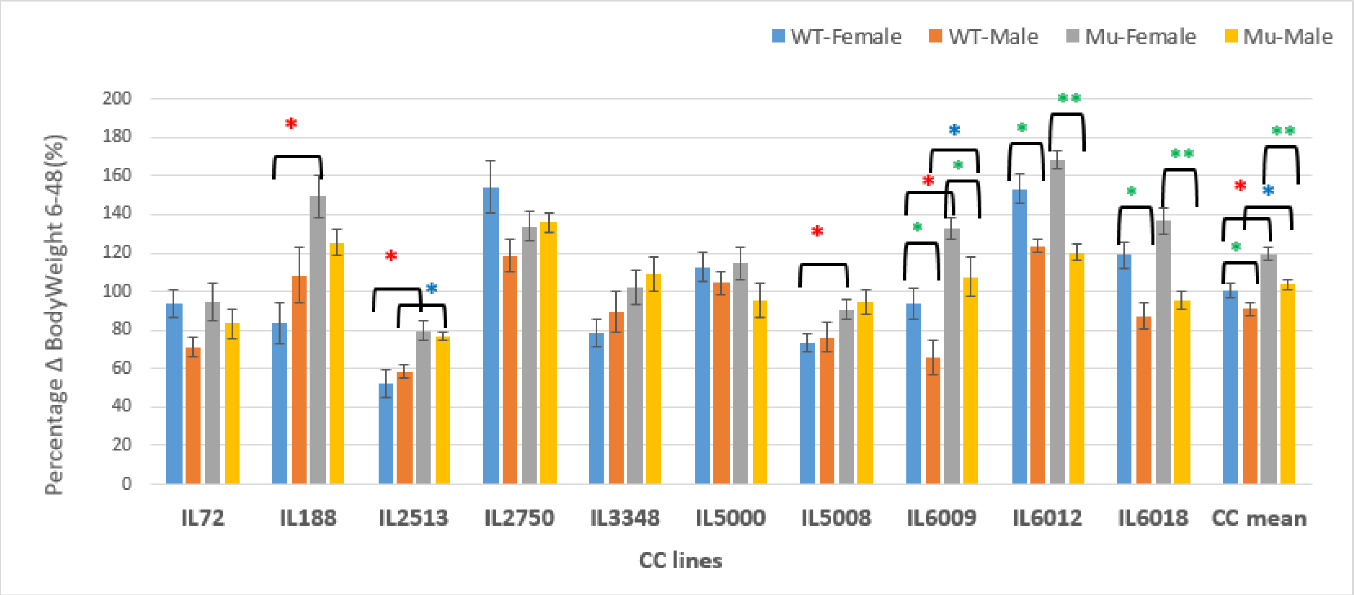

Figure S6. Changes in BW as a percentage in grams between weeks 6-48 of ten CC lines, separately for females and males, having wildtype and mutant genotypes. The X-axis depicts the various CC lines, while the Y-axis depicts the percentage change in BW over weeks 6-48.
